# Supplementary material for: Temporal uncertainty enhances suppression of neural responses to predictable visual stimuli
Source: Neuroimage. 2021 Oct 1;239:118314. doi: 10.1016/j.neuroimage.2021.118314 (PMC8363941; doi:10.1016/j.neuroimage.2021.118314)
Supplement: Supplementary file 1 [file mmc1.docx]

**Supplementary figures:**

**
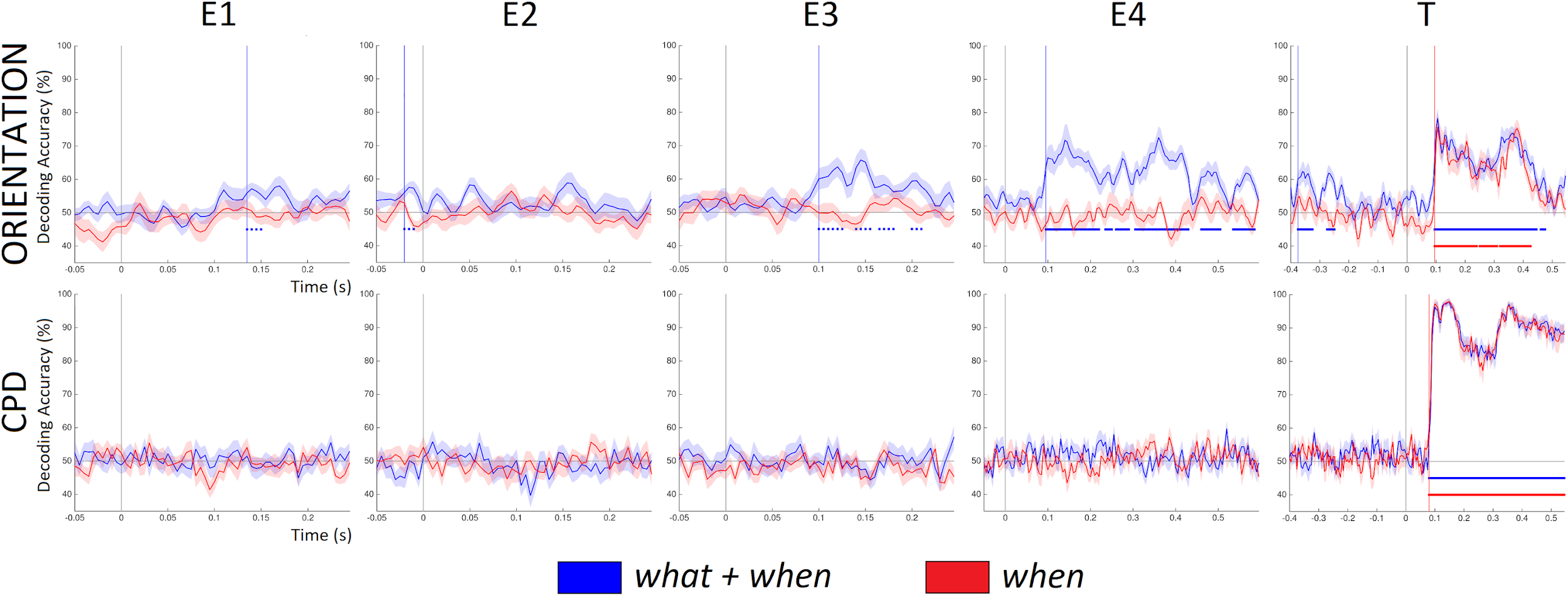
**

**Supplementary Figure 1: Time resolved decoding of *what*+*when* and *what* trials time-locked to Entrainer 1(E1), Entrainer 2 (E2), Entrainer 3 (E3), Entrainer 4 (E4) and Target (T). The coloured dots represent the statistical significance of accuracy.**


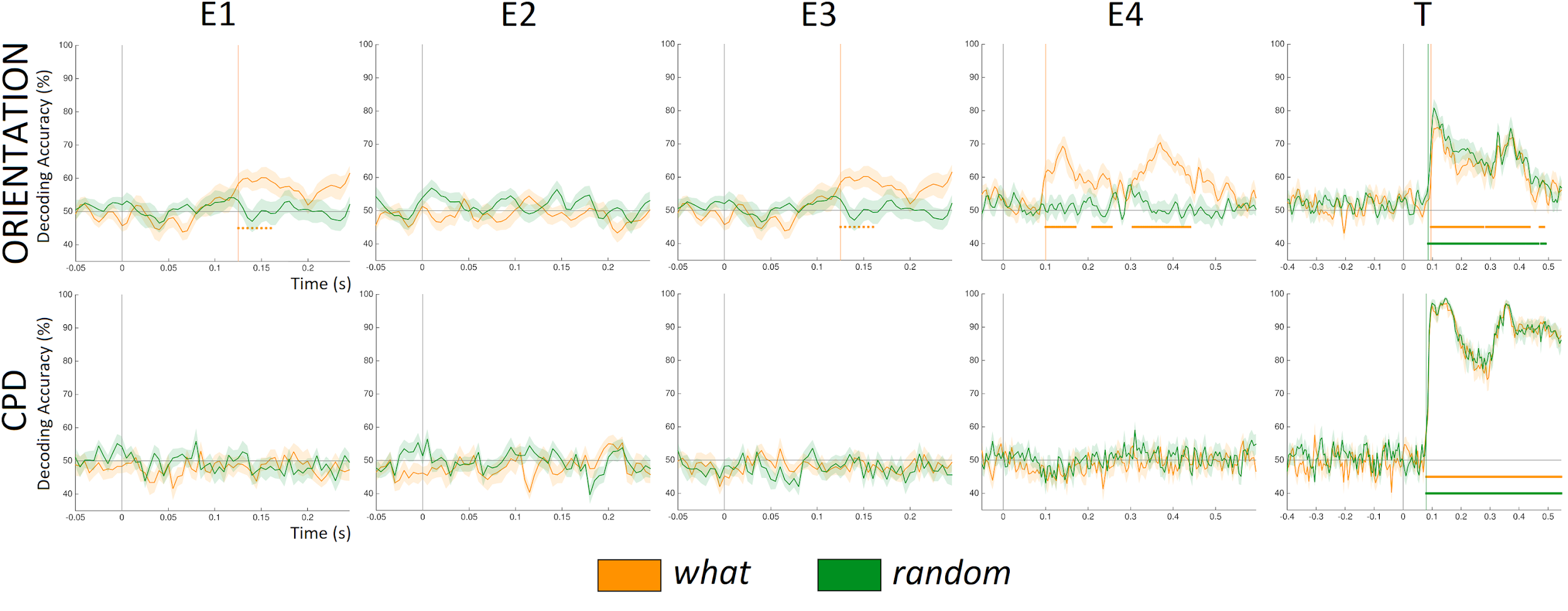


**Supplementary Figure 2 : Time resolved decoding of *what* only and *random* trials time-locked to Entrainer 1(E1), Entrainer 2 (E2), Entrainer 3 (E3), Entrainer 4 (E4) and Target (T). The coloured dots represent the statistical significance of accuracy.**

**Supplementary Table 1**

| **Feature** | **Condition** | **E1** | **E2** | **E3** | **E4** | **Target** |
| --- | --- | --- | --- | --- | --- | --- |
| **Orientation angle** | *what+when* | 135 - 150 ms (56.82 %) | -20 - -10 ms  (57.41 %) | 100 - 125 ms  (63.07 %) | 95 - 215 ms  (70.46 %) | 95 - 450 ms  (78.95 %) |
|  | *when* | ** | ** | ** | ** | 95 - 240 ms (76.84 %) |
|  | *what* | 140 - 165 ms  (56.88 %) | ** | 125  - 160 ms  (60.20 %) | 100 - 170 ms  (68.32 %) | 95 - 275 ms (75.29 %) |
|  | *random* | ** | ** | ** | ** | 85 - 465 ms (75.39 %) |
| **Cycles per degree (CPD)** | *what+when* | ** | ** | ** | ** | 80 - 550 ms (97.73 %) |
|  | *when* | ** | ** | ** | ** | 80 - 545 ms (97.92 %) |
|  | *what* | ** | ** | ** | ** | 85 - 550 ms (97.19 %) |
|  | *random* | ** | ** | ** | ** | 80 - 545 ms (98.65 %) |

** : No Significant cluster found.
